# Participation pattern of methadone users and its association with social connection and HIV status: Analyses of electronic health records data

**DOI:** 10.1371/journal.pone.0216727

**Published:** 2019-05-09

**Authors:** Tsz Ho Kwan, Ngai Sze Wong, Shui Shan Lee

**Affiliations:** 1 Jockey Club School of Public Health and Primary Care, The Chinese University of Hong Kong, Shatin, Hong Kong; 2 Stanley Ho Centre for Emerging Infectious Diseases, The Chinese University of Hong Kong, Shatin, Hong Kong; University of Oxford, UNITED KINGDOM

## Abstract

**Background:**

HIV spread in injecting drug users (IDU) occurs efficiently between individuals within their social networks. While methadone maintenance treatment has long known to be effective in combating HIV transmission in IDU, the impacts of one’s social connections and HIV status have not been well characterised. A study was conducted with the objective of differentiating the pattern of treatment participation between HIV-positive and negative methadone users and to understand its association with social connections with peers.

**Methods:**

Attendance data in one calendar year were extracted from a territory-wide electronic clinical record database of over 8000 methadone users attending 19 clinics in Hong Kong, a city with a relatively low HIV prevalence in injecting drug users. A case-control design was used by matching HIV positive methadone users with HIV negative controls. A temporal-social co-occurrence approach was adopted to construct a social network. Multiple logistic regression and network-based analyses were conducted.

**Results:**

In 2016, a total of 8332 methadone users had attended a clinic at least once, giving 1694016 attendance records that were included in the study. Some 432 methadone, 54 of whom HIV positive, were included in the case-control analyses. Multivariable logistic regression model showed that HIV-positive status was associated with drug injection history (adjusted odds ratio [aOR] 2.28, 95% confidence interval [95% CI] 1.19–4.38), not working fulltime (aOR 3.34, 95% CI 1.15–9.72), ethnic minority (aOR 2.59, 95% CI 1.33–5.02) and minimum daily dose of at least 20mg (aOR 3.64, 95% CI 1.08–12.26). Those having connections with other peers were older (aOR 1.02, 95% CI 1.00–1.04), had a higher mode dose (aOR 1.03, 95% CI 1.02–1.04) and had been admitted to methadone programme for longer time (aOR 1.07, 95% CI 1.02–1.13). Among those with connections, HIV-negative users did not have more connections (median degree centrality 21.00 vs 34.50, p = 0.26) but the network structure was stronger (clustering coefficient 0.65 vs 0.53, p = 0.03).

**Conclusion:**

The weak and sparse linkages may explain the generally low HIV prevalence and incidence in opioid-dependent persons in Hong Kong. Social support could play a constructive role in harm reduction and ethnic minority community-based organisations could help and reinforce treatment adherence.

## Background

Opioid dependence is a multifaceted social and public health issue. Opioid users often resort to injection and may share needles, who are therefore at higher risk of blood-borne infections with HIV and hepatitis C virus (HCV). The global prevalence of HIV and HCV-antibody positive status among injecting opioid users was high at 17.8% and 52.3%, as of 2017.[1] Opioid itself is addictive and causes withdrawal symptoms if discontinued. From a social perspective, opioid dependent users commonly suffer loss of ability to work, resulting in financial difficulty to keep up the drug habit, which in turn induces crimes such as robbery, burglary and shoplifting. Methadone maintenance treatment (MMT) is currently one important means to combat the worldwide problem of opioid dependence. The WHO model list of essential medicines has included methadone since 2005, highlighting the public health threat brought on by opioid addiction.[2]

In Hong Kong, a methadone treatment programme was launched in the mid-1970s to primarily reduce drug-related crimes.[3] The adopted model has continued to be characterized by a low-threshold approach which enables opioid users to have easy access of daily supervised methadone.[4] It was estimated that over 90% of opioid users in the territory were receiving methadone.[5] In 2017, there were 19 methadone clinics in Hong Kong serving over 5000 users.[6] In the same year, only six out of 681 (0.88%) newly reported HIV infections were injecting drug users (IDU).[7] The relatively low HIV prevalence in IDU is speculated to be attributed to the high coverage of MMT.[8] The challenge to the programme in Hong Kong, as is the case anywhere, is to achieve sustainable treatment outcome. Adherence is knowingly the key to such achievement. For HIV-positive users, being on methadone carries health benefit and has been shown to reduce the risk of death.[9] Their high attendance could reduce injection and the subsequent risk of secondary HIV transmission.

Distinct characteristics of methadone users by their serostatus were described in the literature, including age, marital and socioeconomic status.[10] However, the variation in programme participation between HIV-positive and negative users and its association with their social network has yet to be determined. The relationship between methadone users, however, can be difficult to establish specifically. In data mining studies, a spatio-temporal co-occurrence approach has been developed for mining relationships on the frequency of individuals occurring together over space and time.[11] A previous study showed that co-occurrences were correlated with social ties [12]. The higher frequency of such co-occurrences, the more likely links were established, and the stronger their strengths of linkages.[13] An electronic centralised database on methadone users has been in operation since 2007 in Hong Kong recording their clinic attendance thereby enabled relationship mining for social network construction. An analysis of data on methadone treatment adherence in the setting of a low HIV prevalence city could improve our understanding of the relationship between methadone treatment adherence and HIV serostatus. We hypothesised that, HIV-positive methadone users were less socially linked with other users and were not positioned centrally in the social network, otherwise their needle-sharing behaviours would have caused transmission and a higher HIV prevalence in the IDU community. Against these backgrounds, this study aimed to differentiate MMT participation pattern between HIV-positive and negative users and assess its association with social connections with other users, through analysing data accessed from the centralised methadone clinic attendance record database in Hong Kong.

## Methods

### Data

In this study, attendance and admission records of all methadone clinics in Hong Kong in the calendar year of 2016 were accessed. The retrieved data included ethnicity, gender, year of birth and history of injection at first ever admission to the programme. Each entry of the attendance record contained visit date, time, clinic and dose prescribed. If a user has discontinued the use of methadone for 28 days consecutively, he or she would be asked to “readmit” to the programme. At first admission and every readmission thereafter, users’ residential and employment status were collected. Admission records also included the dates of each admission and dropout, and the corresponding clinic attended. Annual HIV test results from voluntary urine specimen collections were retrieved and analysed. Data access approval was obtained from the Department of Health, Hong Kong Special Administrative Region Government. Ethical approval was obtained from The Joint Chinese University of Hong Kong—New Territories East Cluster Clinical Research Ethics Committee (CREC Ref. No.: 2014.613). Informed consent was waived because of the retrospective nature of the study and analysis of anonymous data.

### Study design

One key objective of the study was to distinguish the participation pattern of methadone users by one’s HIV status. HIV-positive users included in the analyses had a positive result before the year 2016. As the prevalence of HIV in methadone users in Hong Kong was low at 1.13%,[14] a case-control design was adopted, with HIV-positive cases and HIV-negative controls randomly matched by first admission year and clinic at a ratio of 1:7 for a higher statistical power.[15] To echo the objective of characterising social connection among methadone users, a network approach was adopted. Users who had connections with another user were compared with those with no connections. Network metrics were compared among methadone users who were connected.

### Network construction algorithm

A spatio-temporal co-occurrence model was developed based on previous works [11,16]. Each clinic attendance of methadone users is an undirected actor-event link which can form a two-mode affiliation network. Co-occurrence of methadone users can therefore be used as the definition of social connection if it is strong enough. The goal of the network construction problem is to extract possible links between two users given a database of their clinic attendance history. The formal definition of the question is as follow: Let *D* be a spatio-temporal database in which each entry is a tuple containing a user’s identifier (*a*), time (*t*) and clinic (*s*) of attendance (Eq 1). Analogous to the notation in a previous work [11], for each tuple, *d*.*a*, *d*.*t* and *d*.*s* denotes a user *d*.*a* attended clinic *d*.*s* at time *d*.*t*.

d=〈a,t,s〉∈D (1)

To be considered as a candidate link in the multiset *G*_*Ecand*_ allowing multiple instances of tuples, it should fulfil both spatial and temporal conditions: both user *i* and user *j* should occur at the same clinic (*d*.*s*) and the difference in time of both occurrence (*d*.*t*) should be within *δ*_*max*_ units of time (Eq 2).

$$G_{Ecand}=\langle d_{i}.a,d_{j}.a\rangle \;|\;(\forall d_{i},d_{j}\in D.A⋀d_{i}.s=d_{j}.s⋀|d_{i}.t-d_{j}.t|<\delta_{max}⋀i\neq j)$$ (2)

Candidate links are not valid unless their frequency is high enough. Let *a*_*i*_ and *a*_*j*_ be user *i* and *j*, respectively, and 〈*a*_*i*_, *a*_*j*_ 〉 be a candidate link between users *i* and *j*. There are two thresholds to be met to be regarded as valid: minimum frequency the link was mined (*w*_*min*_) and the minimum proportion of such occurrence for both users in the link (*p*_*min*_) (Eqs 3 and 4).

$$p_{i|i,j}≔\frac{\left|\langle a_{i},a_{j}\rangle \in G_{Ecand}\right|}{\left|a_{i}\in D.A\right|}$$ (3)

$$p_{j|i,j}≔\frac{\left|\langle a_{i},a_{j}\rangle \in G_{Ecand}\right|}{\left|a_{j}\in D.A\right|}$$ (4)

The links in the resultant social network are in the set *G*_*E*_ (Eq 5) while users in the network are given by *G*_*V*_ (Eq 6).

$$G_{E}=\{\langle a_{i},a_{j}\rangle |p_{i|i,j}\geq p_{min}⋀p_{j|i,j}\geq p_{min}⋀|\langle a_{i},a_{j}\rangle |\geq w_{min}\}$$ (5)

$$G_{V}=\{a|\exists \langle a_{i},a_{j}\rangle \in G_{E},\;a∋\{a_{i},a_{j}\}\}$$ (6)

**Fig 1:**
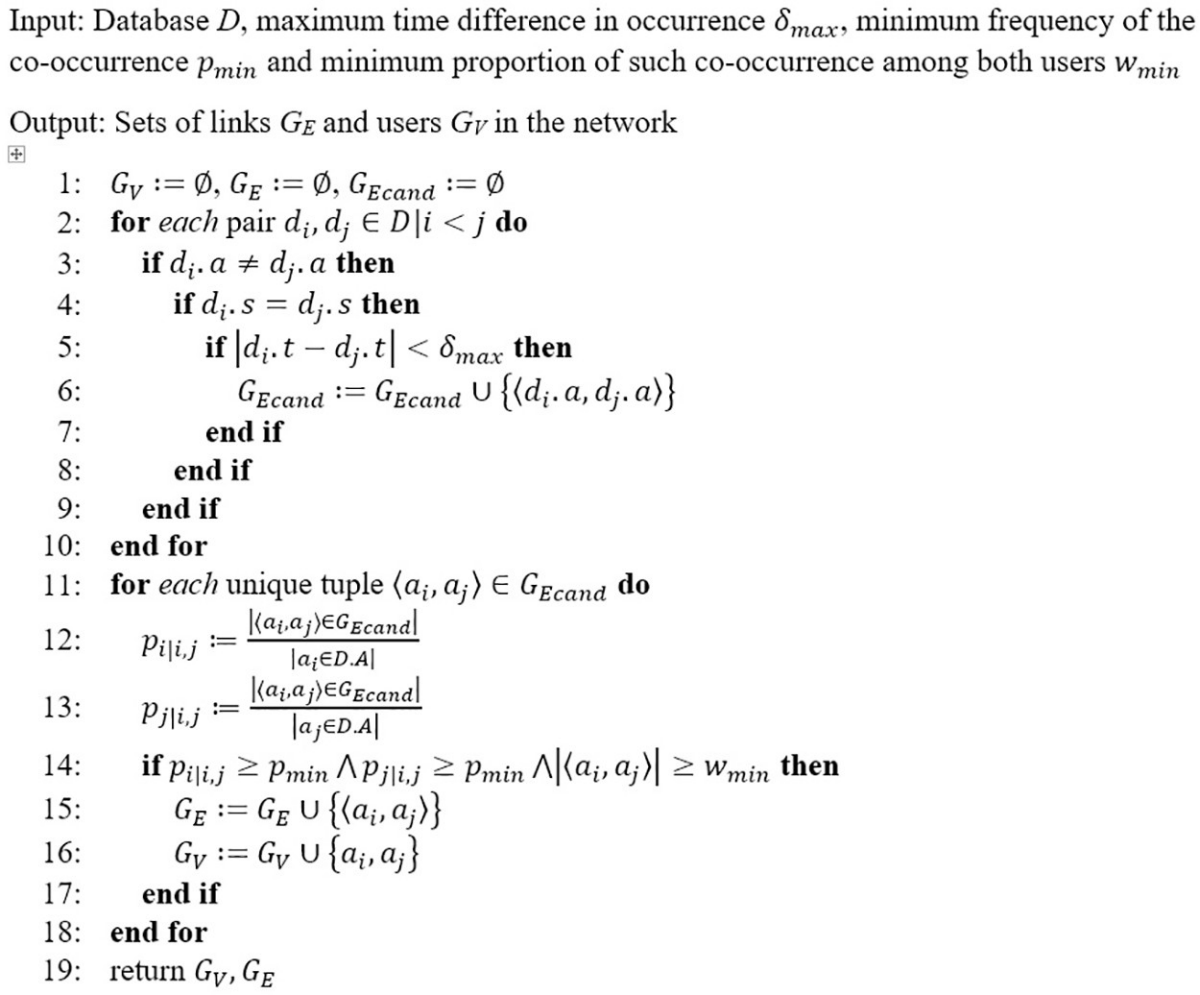
Algorithm for spatial-temporal social network construction.

All possible links fulfilling both the spatial and temporal constraints were stored in the candidate set. After completing the candidate set, links fulfilling the occurrence criteria were included in the resultant network (Fig 1).

In this analysis, we used the attendance records of methadone users in the year of 2016 and defined *δ*_*max*_ as 15 minutes, *p*_*min*_ as 10 and *w*_*min*_ as 10%. That means each link was defined by one’s attendance at the same clinic as another user within 15 minutes of the visits for a minimum of 10 times and accounting for at least 10% of both users’ attendance in a calendar year (2016). As resultant links must fulfil both *p*_*min*_ and *w*_*min*_ constraints, only methadone users who had attended clinic for at least 100 (*w*_*min*_ divided by *p*_*min*_) times in the year were included.

### Variables

Methadone users’ overall participation pattern was assessed by number of years since first admission to the programme and the following attributes in the year of 2016: number of readmissions to the programme after episodes of discontinuation of over 28 days, number of days with clinic visit, and the dose of methadone received.

### Statistical analysis

Comparison was made between methadone users who had connections with at least one other user and those who had not. Among users with connections, degree centrality and clustering coefficient were calculated. Degree centrality was measured to denote the number of a user’s connections while clustering coefficient was used to measure one’s closeness within the local network by the proportion of possible edges present among a user’s neighbours. With a range from 0 to 1, a higher clustering coefficient means the node had closer connections with other nodes. Continuous and discrete variables were tested by Mann-Whitney U and chi-squared or Fisher exact tests, respectively. Variables with p<0.10 were used to construct a multivariable logistic regression model. The variable with the lowest significance of change if removed would be excluded from the model until all variables gave a significance of change of less than 0.10. For assessing predictors of social connections, number of visits and readmissions in 2016 were excluded from the equation as these two were confounded in the definition of connections. Statistical analysis was performed with SPSS Version 25.

## Results

**Fig 2:**
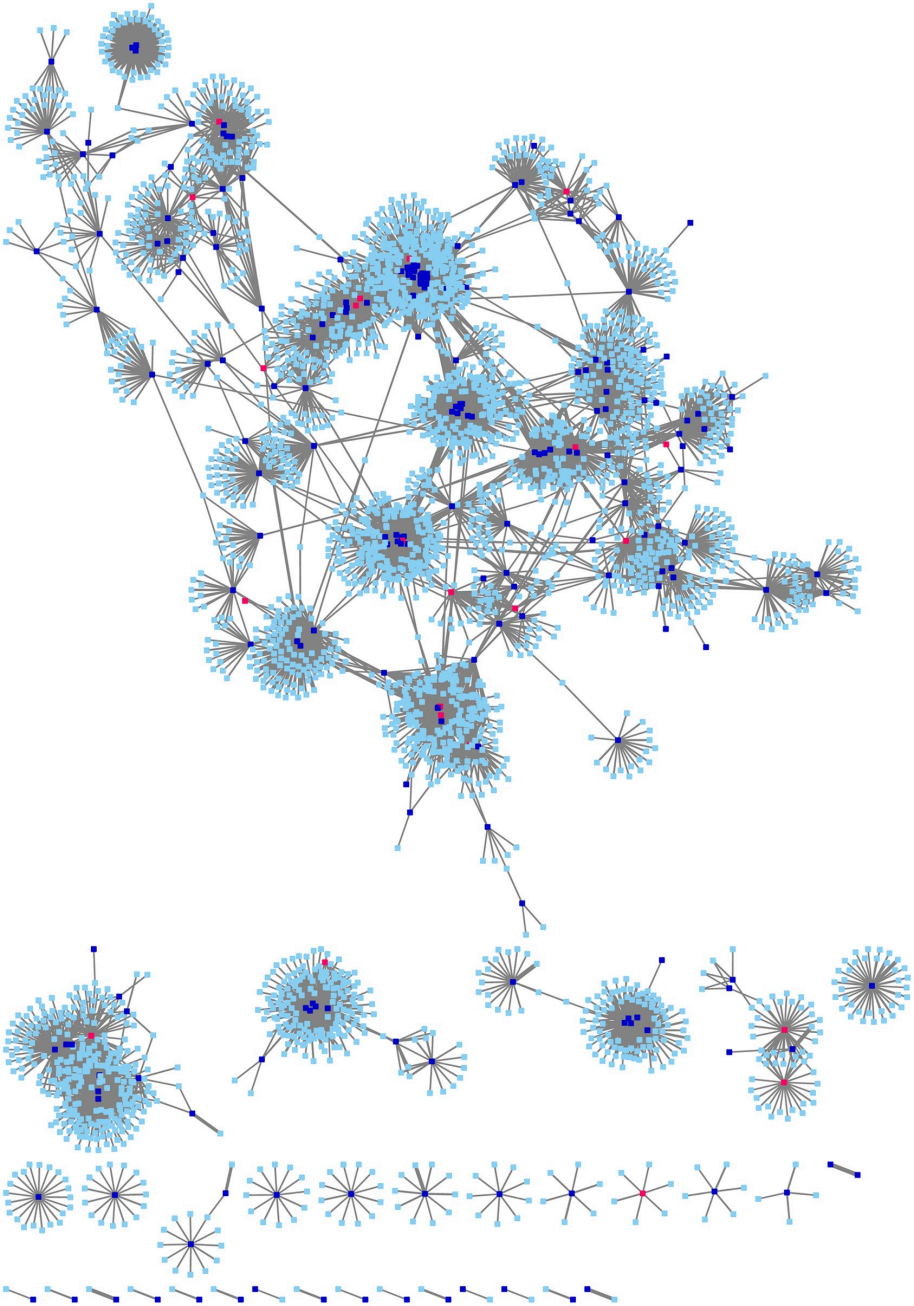
Social connections between methadone users.

In 2016, a total of 8425 methadone users had attended a methadone clinic at least once, of which 93 escorted by law enforcement officers were excluded from the analyses. Totally 8332 users with 1694016 attendance records were included in the analyses. Some 69% (5716/8332) of users had at least one connection with another user, forming a total of 130024 connections. Among connected users, some 5687 (99%) had at least one HIV test in the records over the years, with 57 (1%) giving positive results. Almost all (54/57) HIV-positive methadone users could each match with 7 HIV-negative controls by the same admission year and clinic. A total of 432 users were therefore included in the analyses. Overall, the median duration since first admission to MMT was 9 years (inter-quartile range [IQR]: 1–11 years). In 2016, they attended a median of 134 days (IQR: 31–298 days) with a median of 2 readmissions (IQR: 0–4). The median of mode of methadone dose received was 40mg (IQR: 30–54 mg). The medians of minimum and maximum were respectively 30mg and 50mg (IQR: 20–44 mg; 35-70mg). Some 77 (18%) had been prescribed a minimum dose of less than 20mg in 2016. Half of the users had a dose range of 15 mg (IQR: 5-30mg) within the same year. Over half (57%, 246/432) had at least one connection with another methadone user. Among those connected with other users, they had a median of 24 links (IQR 7–71) with a median clustering coefficient of 0.65 (IQR: 0.43–0.80). Fig 2 shows the social connections between cases, controls, and their directly connected users not included in the case-control analysis.

Only HIV-positive cases (coloured in red) their controls (coloured in blue), and all their directly connected neighbours (coloured in cyan) were shown.

Ethnic Chinese accounted for 89% of all analysed cases. HIV-positive methadone users were less likely ethnic Chinese (odds ratio [OR] 0.50, 95% confidence interval [95% CI] 0.28–0.90, p = 0.02), and had history of injection before admission (OR 2.16, 95% CI 1.19–3.93, p = 0.01) (Table 1). Of note, ethnic minority methadone users were more likely drug injection users before their first admission (OR 3.14, 95% CI 1.83–5.39, p<0.001). HIV-positive methadone users also, at the first admission, had higher odds of being unemployed (OR 2.09, 95% CI 1.13–3.88, p = 0.02) and not working fulltime (OR 0.31, 95% CI 0.11–0.89, p = 0.02). They were also younger (median age 43 vs 47 years) and first admitted at a younger age than HIV-negative ones (median age 36 vs 41 years, p = 0.01). However, the length, as controlled, and strength of methadone use did not show significant difference. Adherence, as inferred from the total number of temporary discontinuation from the programme and number of visits in 2016, did not differ between HIV-positive and negative users. HIV-negative users had higher odds of having received the lowest dose of less than 20 mg in 2016 (OR 0.24, 95% CI 0.07–0.80, p = 0.01). In term of social connection, HIV-positive users were less likely to be connected with another methadone user (OR 0.47, 95% CI 0.27–0.84, p = 0.01). Multivariable logistic regression showed that, a history of injection at first admission (adjusted odds ratio [aOR] 2.28, 95% CI 1.19–4.38, p = 0.01) was a predicting variable of seropositive methadone users. Working fulltime (aOR 0.30, 95% CI 0.10–0.87, p = 0.03) at first admission, being ethnic Chinese (aOR 0.39, 95% CI 0.20–0.75, p = 0.01) and having had a minimum dose of less than 20mg (aOR 0.28, 95% CI 0.08–0.93, p = 0.04) were negative predictors.

**Table 1:** Characteristics of case-controlled HIV-positive and negative users who attended the methadone programme in 2016 (N = 432).

	HIV-positive methadone users (N = 54)	HIV-negative methadone users (N = 378)			
	n (%) *median (IQR)*	n (%) *median (IQR)*	OR (95% CI)	X^2^*U*	P
Demographics					
Ethnic Chinese	33 (61%)	287 (76%)	0.50 (0.28–0.90)	5.40	0.02
Male	50 (93%)	346 (92%)	1.16 (0.39–3.41)	-	0.52^a^
Age in 2016, years	*43 (38–48)*	*47 (36–59)*	-	*8449*	0.04
Attributes at first enrolment					
Age, years	*36 (32–42)*	*41 (31–49)*	-	*7988*	0.01
Working fulltime	4 (7%)	77 (20%)	0.31 (0.11–0.89)	5.21	0.02
Working parttime	8 (15%)	42 (11%)	1.39 (0.62–3.15)	0.63	0.43
Being unemployed	38 (70%)	201 (53%)	2.09 (1.13–3.88)	5.65	0.02
Having a history of injection	25 (50%)	107 (32%)	2.16 (1.19–3.93)	6.53	0.01
Methadone programme utilisation pattern					
Number of re-admissions in 2016	*2 (1–5)*	*2 (0–4)*	-	*9146*	0.21
Number of clinic visits in 2016	*91 (12–245)*	*145 (32–303)*	-	*8664*	0.07
Mode dose in 2016, mg	*40 (30–51)*	*40 (30–55)*	-	*9894*	0.71
Minimum dose in 2016, mg	*30 (20–40)*	*30 (20–40)*	-	*10189*	0.98
Minimum dose <20 mg in 2016	3 (6%)	74 (20%)	0.24 (0.07–0.80)	6.34	0.01
Maximum dose in 2016, mg	*45 (39–70)*	*50 (35–70)*	-	*9929*	0.75
Dose range in 2016, mg	*15 (5–30)*	*18 (5–30)*	-	*9984*	0.79
Social connections with other users					
Connected with at least one other user in 2016	22 (41%)	224 (59%)	0.47 (0.27–0.84)	6.61	0.01
Degree centrality (N = 22; 224)	*34*.*50 (9*.*75–75*.*50)*	*21*.*00 (6*.*25–68*.*00)*	-	*2105*	0.26
Clustering coefficient (N = 22; 224)	*0*.*53 (0*.*35–0*.*67)*	*0*.*65 (0*.*46–0*.*81)*	-	*1773*	0.03

^a^ Fisher’s exact test

Compared with unconnected methadone users, users who were connected with another user were more likely ethnic Chinese (OR 2.52, 95% CI 1.62–3.91, p<0.001), and less likely unemployed at first admission (OR 0.52, 95% CI 0.35–0.76, p = 0.001) (Table 2). The injection history was not significantly different between the two groups. Those connected with another user were older (p<0.001) and had been admitted to the programme for a longer period of time (p<0.001). Their adherence in MMT was comparatively higher, as reflected by their lower number of discontinuation from the programme (1 vs 2, p = 0.03) and higher number of attendance (73% vs 8%, p<0.001) during the one-year period. In term of dosage of methadone, users with such connections took a higher dose (p<0.001) yet its range within the year was also higher (20mg vs 10mg, p = 0.002). Being HIV-positive (aOR 0.49, 95% CI 0.26–0.94, p = 0.03) and being unemployed at first admission (aOR 0.58, 95% CI 0.38–0.90, p = 0.01) were predictors of not having social connections with any other methadone users. Older age (aOR 1.02, 95% CI 1.00–1.04, p = 0.04), higher mode dose of methadone (aOR 1.03, 95% CI 1.02–1.04, p<0.001), and admission for a longer period (aOR 1.07, 95% CI 1.02–1.13, p = 0.01) were predictors of being connected in the network. Among methadone users who were connected with at least one peer, HIV-positive users did not have significantly more connections than HIV-negative users (median degree centrality 34.50 vs 21.00, p = 0.26), but the clustering coefficient was lower (0.53 vs 0.65, p = 0.03).

**Table 2:** Comparison between methadone users active in 2016 with and without social connections with another user (N = 432).

	Connected (N = 246)	Not connected (N = 186)			
	n (%) *median (IQR)*	n (%) *median (IQR)*	OR (95% CI)	X^2^*U*	P
Demographics					
Ethnic Chinese	201 (82%)	119 (64%)	2.52 (1.62–3.91)	17.34	<0.001
Male	227 (92%)	169 (91%)	1.20 (0.61–2.38)	0.28	0.60
Age in 2016, years	*52 (40–61)*	*41 (34–50)*	-	*15403*	<0.001
Attributes at first enrolment					
Age, years	*43 (34–50)*	*37 (30–44)*	-	*17197*	<0.001
Working fulltime	53 (22%)	28 (15%)	1.55 (0.94–2.57)	2.93	0.09
Working parttime	22 (9%)	28 (15%)	0.55 (0.31–1.00)	3.86	0.049
Being unemployed	119 (48%)	120 (65%)	0.52 (0.35–0.76)	11.17	0.001
Having a history of injection	64 (32%)	68 (37%)	0.79 (0.52–1.21)	1.18	0.28
Methadone programme utilisation pattern					
Length of methadone use, years	*10 (3–11)*	*3 (0–10)*	-	*14598*	<0.001
Number of re-admissions in 2016	*1 (0–3)*	*2 (0–5)*	-	*20190*	0.03
Number of clinic visits in 2016	*267 (155–352)*	*28 (8–65)*	-	*3098*	<0.001
Mode dose in 2016, mg	*40 (35–66)*	*35 (25–40)*	-	*15030*	<0.001
Minimum dose in 2016, mg	*35 (20–40)*	*30 (20–40)*	-	*17070*	<0.001
Minimum dose <20 mg in 2016	41 (17%)	36 (19%)	0.83 (0.51–1.37)	0.52	0.47
Maximum dose in 2016, mg	*55 (40–75)*	*40 (30–55)*	-	*14406*	<0.001
Dose range in 2016, mg	*20 (5–35)*	*10 (0–25)*	-	*18872*	0.002

## Discussion

In this study we applied a spatial-temporal co-occurrence network approach to understand the participation pattern of opioid users registered with a low threshold methadone treatment programme in Hong Kong, using data accessed from a centralised electronic database. We have shown that HIV-positive methadone users had distinct characteristics in socioeconomic status and service utilisation pattern compared to seronegative users in the same year and at the same clinic. While 92% of the population were ethnic Chinese,[17] some 14% of the methadone users were non-Chinese ethnic minority, most of whom Nepali, Vietnamese and other South Asians.[18] Our results revealed that non-Chinese methadone users were more likely to be HIV-positive. A previous study has shown that HIV knowledge of non-Chinese drug users in Hong Kong was poor, which might explain a higher tendency of engaging in high risk behaviours, including needle sharing and condomless sex, leading to virus transmission.[19] From our results, we also observed higher odds of drug injection history at first admission in the ethnic minority. HIV-positive methadone users were, on the other hand, more likely to be unemployed at first admission. Unemployment among HIV-positive persons could be due to frailty and disease severity while strong dependence on drug use rendered them unable to work.[20] HIV-positive methadone users may therefore have faced extra difficulties en route to recovery. They were, in general, younger and have been admitted to MMT at a younger age, but the length of methadone use was not longer. It can be inferred that HIV-positive users may have started the habit of drug use at a younger age. The dose they took was not significantly higher than HIV-negative one, but smaller proportion of them took a minimum dose of less than 20mg. The low methadone dose may reflect the situation of some users in the process of going for abstinence progress, but such treatment may not be effective enough to suppress withdrawal symptoms. They may return to their drug habit which put them at risk of attaining poorer health outcome.[21]

Our results suggested that the social connectivity of methadone users differed by their HIV status. HIV-positive methadone users were less likely to have social connections with their peers, and even if they did, the strength of the social bonding was comparatively weaker compared to their HIV-negative counterparts. Determinants of such connections included employment status, age, and length and dose of methadone use. This would suggest that it took time to build up connections with peers. Longer methadone use and older age were therefore predictors of the presence of connections. Users working at similar hours may take methadone at similar time, thus be able to meet their peers and provide opportunity to build up social bonds. Ethnicity was also associated with social connections, as reported previously that, ethnic minorities preferred to build racial homophilic connections.[19] Yet such social relationship can be a double-edged sword—emotional support from network members could offer emotional support which in turn reduced heroin use and encouragement and discussion on HIV risks with peers would reduce engagement in sexual risk behaviours; [22–23] on the other hand, such relationships may facilitate needle sharing practice,[24] thereby hampering the effort of harm reduction and HIV prevention.

HIV prevention is an important outcome of methadone treatment. Overseas studies have reported that methadone users receiving higher doses could achieve higher adherence in antiretroviral treatment for HIV.[25] In our study the finding of a lower proportion of HIV-positive methadone users taking low methadone doses may have resulted from the support of healthcare workers at HIV services they attended simultaneously. This is in line with the WHO recommendation of maintaining a higher dose of methadone in HIV-positive users, realising that both antiretroviral and opioid substitution therapy were important components of a comprehensive harm reduction package.[26] Receiving higher doses also represented one’s reliance on methadone, may as well contributed to a more stable lifestyle that they were able to engage in employment. Promotion of adherence to an optimal dose of methadone is crucial for achieving sustained outcome. Self-support, such as setting mobile phone alarms, and social support from family and peers were means to foster treatment adherence.[27–28] These support could also reduce opioid users’ self-perceived stigma and improve mental well-being.[29] On the other hand, one’s age and education level could be associated with methadone programme utilisation.[30]

Social instability and HIV risk behaviours may have formed a feedback loop reinforcing drug injection and needle sharing practice, thereby predisposing to HIV infection before accessing methadone. A higher proportion of HIV-positive users maintained on methadone compared with HIV-negative users was an important observation that carries public health benefits. It appears that formation of social network was founded on one’s methadone utilisation pattern and socioeconomic status. HIV-positive users in this study were sparsely connected with other methadone users. The weak or absence of social connections with HIV-positive users may explain the low HIV transmission among the injection drug user community. The adherence of HIV-positive methadone users was not worse than HIV-negative ones, therefore social bonding was not the only factor contributed to the maintenance in the programme but external factors, such as poorer health status, being incarcerated, and living further from clinic,[31–32] were likely in place.

This study has several limitations. Firstly, the attendance history in the database only included the time of dispensing methadone for individual user, not the duration of staying in the clinic. Some methadone users in Hong Kong stayed in gathering places near the clinics with their peers,[8] social connections could therefore happen there instead of the short interval inside the clinic defined in this study. These linkages between users could not be reflected in the analysis. On the other hand, the presence of relationship between users may not reflect their true linkage in real life although co-occurrences and actual social connections could be strongly correlated [12]. Secondly, timing of HIV seroconversion has not been taken into account in the analysis. The temporal relationship between HIV transmission and the methadone use was not known, and could only be inferred from the participation pattern of methadone users as differentiated by their HIV status. Thirdly, as HIV prevalence among methadone users was low, case-control analysis was adopted. Causal relationships cannot not be established from the results. However, with the use of comprehensive electronic clinic attendance records, we were able to infer social connections, to characterise the attendance pattern and to distinguish HIV-positive users from their baseline attributes and participation intensity.

In summary, high HIV risk behaviours and unstable income source may have predisposed injecting drug users to HIV infection before admitting to methadone programme in Hong Kong. The higher HIV prevalence of ethnic minority methadone users is a cause for concern as regards HIV transmission risk. The sparse and weak social connections between users might explain partly the low HIV prevalence and incidence in the territory. Social support, however, could play a constructive role in harm reduction as seen from their longer duration of retention in programme and higher dose. Community efforts could fill the gap in social connections and contribute to the achievement of sustainable treatment outcome of methadone maintenance.

## References

[1] Degenhardt L, Peacock A, Colledge S, Leung J, Grebely J, Vickerman P, et al Global prevalence of injecting drug use and sociodemographic characteristics and prevalence of HIV, HBV, and HCV in people who inject drugs: a multistage systematic review. Lancet Glob Health 2017;5(12):e1192–e1207. 10.1016/S2214-109X(17)30375-3 29074409 PMC5683738

[2] WHO Model List of Essential Medicines. http://www.who.int/medicines/publications/essentialmedicines/en/ Accessed January 7, 2019.

[3] Lee SS, Newman RG. Methadone maintenance—lessons from two systems in China. Harm Reduct J 2017;14:66 10.1186/s12954-017-0193-7 28946906 PMC5613321

[4] Newman RG. Globally informed, locally responsive. New York: Open Society Foundations 2017.

[5] Wong NS, Chan PC, Lee SS, Lee SL, Lee CK. A multilevel approach for assessing the variability of hepatitis C prevalence in injection drug users by their gathering places. Int J Infect Dis 2013;17(3):e193–8. 10.1016/j.ijid.2012.10.004 23165126

[6] LCQ9: Methadone Treatment Programme. https://www.info.gov.hk/gia/general/201810/31/P2018103100452.htm Accessed January 7, 2019.

[7] HIV/AIDS situation in Hong Kong [2017]. https://www.aids.gov.hk/english/surveillance/sur_report/hiv_fc2017e.pdf Accessed January 7, 2019.

[8] Lee SS. The contribution of methadone maintenance treatment to HIV prevention—the case of Hong Kong [abstract]. Conference proceedings of International Conference on Tackling Drug Abuse. http://www.nd.gov.hk/en/conference_proceedings/Drugs_proBK_Part3/Drugs_proBK_LeeSS.pdf Accessed January 7, 2019.

[9] Zhao Y, Shi CX, McGoogan JM, Rou K, Zhang F, Wu Z. Methadone maintenance treatment and mortality in HIV-positive people who inject opioids in China. Bull World Health Organ 2013;91(2):93–101. 10.2471/BLT.12.108944 23554522 PMC3605005

[10] Yen YF, Yen MY, Lin T, Li LH, Jiang XR, Chou P, et al Prevalence and factors associated with HIV infection among injection drug users at methadone clinics in Taipei, Taiwan. BMC Public Health 2014;14:682 10.1186/1471-2458-14-682 24996558 PMC4098925

[11] Lauw HW, Lim EP, Pang Hm, Tan TT. STEvent: Spatio-temporal event model for social network discovery. ACM Trans Inf Syst. 2010;28(3):15.

[12] Li RH, Liu J, Yu JX, Chen H, Kitagawa H. Co-occurrence prediction in a large location-based social network. Front Comput Sci. 2013;7(2):185–94.

[13] Lauw HW, Lim EP, Pang H, Tan TT. Social Network Discovery by Mining Spatio-Temporal Events. Comput Math Organ Theory. 2005;11(2):97–118.

[14] HIV surveillance report– 2016 update. https://www.aids.gov.hk/english/surveillance/sur_report/hiv16.pdf Accessed January 7, 2019.

[15] Hennessy S, Bilker WB, Berlin JA, Strom BL. Factors influencing the optimal control-to-case ratio in matched case-control studies. Am J Epidemiol 1999;149(2):195–7. 9921965 10.1093/oxfordjournals.aje.a009786

[16] Kwan TH, Wong NS, Lee SS. Participation dynamics of a cohort of drug users in a low-threshold methadone treatment programme. Harm Reduct J 2015;12:30 10.1186/s12954-015-0072-z 26470863 PMC4608158

[17] Women and men in Hong Kong—key statistics. [2018]. Hong Kong: Census and Statistics Department 2018. https://www.statistics.gov.hk/pub/B11303032018AN18B0100.pdf Accessed January 7, 2019.

[18] Review of epidemiology for Ethnic Minorities (EM) in Hong Kong. Hong Kong: Hong Kong Advisory Council on AIDS 2015. http://www.aca.gov.hk/english/strategies/pdf/EM%20Eng.pdf Accessed January 7, 2019.

[19] Mak WL, Lee CK, Wong KH, Ng H, Wong C. HIV knowledge, drug use behaviour and infection status of ethnic minorities drug users attending methadone clinics in Hong Kong [abstract]. Poster session presented at Health Research Symposium 2007; 2007 September 29; Hong Kong. https://www.rrc.gov.hk/research/ab82.pdf Accessed January 7, 2019.

[20] Gross M, Herr A, Hower M, Kuhlmann A, Mahlich J, Stoll M. Unemployment, health, and education of HIV-infected males in Germany. Int J Public Health 2016;61(5):593–602. 10.1007/s00038-015-0750-3 26427862 PMC4947124

[21] Darker CD, Ho J, Kelly G, Whiston L, Barry J. Demographic and clinical factors predicting retention in methadone maintenance: results from an Irish cohort. Ir J Med Sci 2016;185(2):433–41. 10.1007/s11845-015-1314-5 26026953

[22] Shen L, Assanangkornchai S, Liu W, Cai L, Li F, Tang S, et al Influence of social network on drug use among clients of methadone maintenance treatment centers in Kunming, China. PLoS One 2018;13(7):e0200105 10.1371/journal.pone.0200105 29969481 PMC6029801

[23] El-Bassel N, Gilbert L, Wu E, Chang M. A social network profile and HIV risk among men on methadone: do social networks matter?. J Urban Health 2006;83(4):602–13. 10.1007/s11524-006-9075-0 16755389 PMC2430490

[24] Unger JB, Kipke MD, De Rosa CJ, Hyde J, Ritt-Olson A, Montgomery S. Needle-sharing among young IV drug users and their social network members: The influence of the injection partner's characteristics on HIV risk behavior. Addict Behav 2006;31(9):1607–18. 10.1016/j.addbeh.2005.12.007 16459023

[25] Lappalainen L, Nolan S, Dobrer S, Puscas C, Montaner J, Ahamad K, et al Dose-response relationship between methadone dose and adherence to antiretroviral therapy among HIV-positive people who use illicit opioids. Addiction 2015;110(8):1330–9. 10.1111/add.12970 25940906 PMC4503496

[26] WHO, UNODC, UNAIDS technical guide for countries to set targets for universal access to HIV prevention, treatment and care for injecting drug users– 2012 revision. Geneva: World Health Organization 2013. https://www.who.int/hiv/pub/idu/targets_universal_access/en/ Accessed January 7, 2019.

[27] Nguyen LH, Nguyen HTT, Nguyen HLT, Tran BX, Latkin CA. Adherence to methadone maintenance treatment and associated factors among patients in Vietnamese mountainside areas. Subst Abuse Treat Prev Policy 2017;12(1):31 10.1186/s13011-017-0115-4 28595642 PMC5465686

[28] Spohr SA, Livingston MD, Taxman FS, Walters ST. What's the influence of social interactions on substance use and treatment initiation? A prospective analysis among substance-using probationers. Addict Behav 2018;89:143–50. 10.1016/j.addbeh.2018.09.036 30316139 PMC6240372

[29] Birtel MD, Wood L, Kempa NJ. Stigma and social support in substance abuse: Implications for mental health and well-being. Psychiatry Res 2017;252:1–8. 10.1016/j.psychres.2017.01.097 28237758

[30] Lan CW, Lin C, Thanh DC, Li L. Drug-related stigma and access to care among people who inject drugs in Vietnam. Drug Alcohol Rev 2018;37(3):333–9. 10.1111/dar.12589 28762584 PMC5794669

[31] Tran BX, Nguyen LH, Tran TT, et al Social and structural barriers for adherence to methadone maintenance treatment among Vietnamese opioid dependence patients. PLoS ONE 2018;13(1):e0190941 10.1371/journal.pone.0190941 29346444 PMC5773191

[32] Lin CK, Hung CC, Peng CY, Chao E, Lee TS. Factors associated with methadone treatment duration: a cox regression analysis. PLoS ONE 2015;10(4):e0123687 10.1371/journal.pone.0123687 25875531 PMC4397075

